# A Genome-Wide Association Study Revealed Key SNPs/Genes Associated With Salinity Stress Tolerance In Upland Cotton

**DOI:** 10.3390/genes10100829

**Published:** 2019-10-21

**Authors:** Muhammad Yasir, Shoupu He, Gaofei Sun, Xiaoli Geng, Zhaoe Pan, Wenfang Gong, Yinhua Jia, Xiongming Du

**Affiliations:** 1 Institute of Cotton Research of Chinese Academy of Agricultural Sciences (ICR, CAAS)/State Key Laboratory of Cotton Biology, Anyang 455000, Henan, China; yasir1735@gmail.com (M.Y.); heshoupu@caas.cn (S.H.); czxiaoli@126.com (X.G.); panzhaoe@163.com (Z.P.); gwf018@126.com (W.G.); jiayinhua_0@sina.com (Y.J.); 2 Anyang Institute of Technology, Anyang 455000, Henan, China; sungaofei@sina.com

**Keywords:** cotton, genome-wide association study (GWAS), SNPs, salinity

## Abstract

Millions of hectares of land are too saline to produce economically valuable crop yields. Salt tolerance in cotton is an imperative approach for improvement in response to ever-increasing soil salinization. Little is known about the genetic basis of salt tolerance in cotton at the seedling stage. To address this issue, a genome-wide association study (GWAS) was conducted on a core collection of a genetically diverse population of upland cotton (*Gossypium hirsutum* L.) comprising of 419 accessions, representing various geographic origins, including China, USA, Pakistan, the former Soviet Union, Chad, Australia, Brazil, Mexico, Sudan, and Uganda. Phenotypic evaluation of 7 traits under control (0 mM) and treatment (150 mM) NaCl conditions depicted the presence of broad natural variation in the studied population. The association study was carried out with the efficient mixed-model association eXpedited software package. A total of 17,264 single-nucleotide polymorphisms (SNPs) associated with different salinity stress tolerance related traits were found. Twenty-three candidate SNPs related to salinity stress-related traits were selected. Final key SNPs were selected based on the r^2^ value with nearby SNPs in a linkage disequilibrium (LD) block. Twenty putative candidate genes surrounding SNPs, A10_95330133 and D10_61258588, associated with leaf relative water content, RWC_150, and leaf fresh weight, FW_150, were identified, respectively. We further validated the expression patterns of twelve candidate genes with qRT-PCR, which revealed different expression levels in salt-tolerant and salt-sensitive genotypes. The results of our GWAS provide useful knowledge about the genetic control of salt tolerance at the seedling stage, which could assist in elucidating the genetic and molecular mechanisms of salinity stress tolerance in cotton plants.

## 1. Introduction

It has been estimated that soil salinity has affected 80 million hectares of the world’s cultivated land [1]. Millions of hectares of land are too saline to produce economically valuable crop yields, and this trend is on the rise every year, making the land nonproductive [2]. The problem of soil salinization is worsening in countries, such as the USA, China, Australia, Hungary and is threatening to become more intense in North and East African countries, and Middle Eastern and East Asian countries. Salt-affected areas in China constitute 4.88% of the country’s total land area, which equates to 3.6 × 10^7^ ha of land nationwide [3]. Excessive salinity of the soil can cause ion toxicity, osmotic stress, water, and nutrient scarcity and, thus, promptly decrease crop growth due to reduced photosynthesis [4]. Ionic homeostasis, balanced root water intake, and leaf transpiration coupled with increased nutrient uptake are crucial for plants to deal with salinity stress [5]. In addition to arid and semiarid areas, which account for approximately 30% of global saline areas, 20% of irrigated land has saline soils, and this proportion continues to increase [6]. Due to environmental degradation caused by climate change, the salinity conundrum will also become more severe in areas with low and mid-latitudes. Cotton is a global industrial crop grown to meet the demand of 7.7 billion people worldwide. The exponential increase in the global population is expected to reach almost 9 billion by 2050 [7], consequently increasing the demand for food and fiber equally. In order to combat future challenges, breeders are trying to improve cotton varieties especially for marginal lands, such as saline and drought-affected soils.

The genetic causes of phenotypic variations aimed at improving crop productivity and biotic and abiotic stress tolerance have been major focuses of plant studies. Quantitative trait locus (QTL) mapping has been successfully used in plants for mapping biparental crosses to detect sections of genomes that co-segregate with a certain trait either in F2 populations or in recombinant inbred line (RIL) families [8,9]. Nevertheless, QTL mapping has two major pitfalls: it is constrained by both allelic diversity and narrow genomic resolution caused by relatively few recombination events that occur during the creation of the RIL population [10]. Genome-wide association studies (GWASs) have the potential to overcome possible major limitations of QTL mapping by providing relatively high resolution even at the gene level, and the use of samples from previously well-studied populations with naturally occurring genetic variants can be concomitant with phenotypic variation. The basic approach in GWASs is to evaluate the association between each genotyped single-nucleotide polymorphism (SNP) marker and a phenotype of interest that has been recorded across a RIL population or a large number of individuals of a natural population. Recent developments in sequencing techniques have enabled GWASs to emerge as the most successful tool for identifying genetic causes of complex quantitative traits in plant species [11]. GWASs have been successfully used to dissect the underpinnings of yield traits [12], salinity, water deprivation, and heavy metal stress tolerance [13,14]; fiber quality traits, and disease resistance [15,16]. GWASs have been successfully used in cotton crops to trace genetic signatures associated with yield parameters and stress tolerance traits [17,18,19], but there is a lack of extensive study in the cotton crop for stress tolerance at the seedling stage [20]. In a previous study conducted by Sun et al. [21], the authors found twenty-three SNPs on seven chromosomes associated with two salt tolerance related traits in cotton.

Cotton (*Gossypium hirsutum* L.) is an important fiber crop species and the only commercial crop where the fiber is converted to fabric at a commercial scale [22]. Salt stress can affect plant growth and development throughout plant ontogeny, but the seedling stage is considered one of the most vulnerable stages [23]. Moreover, although cotton is considered moderately salt-tolerant with a cut-off of 7.7 dS/m, its growth is severely affected at the seedling stage, which reduces the yield [24]. The current study was designed to perform a GWAS of salt tolerance traits associated with 17,264 SNPs in a core collection of 419 diverse natural populations of *G. hirsutum* L. at the seedling stage. The present study pinpoints associated SNPs and probable genes to decipher the complex genetic background of salt stress tolerance in cotton. The principal theoretical implication of this study is the development of molecular markers that will foster salt stress tolerance breeding programs in cotton.

## 2. Materials and Methods

### 2.1. Germplasm Collection

In this study, a core collection of 419 genotypes of upland cotton was used, derived from a recently published study [25]. Out of the 419 genotypes, 317 accessions were collected from different provinces of China and reaming accessions were collected from major cotton-growing countries viz. Australia, Brazil, Bulgaria, Chad, France, Japan, Mexico, Pakistan, Russia, Spain, Sudan, Turkey, Uganda, USA, and Uzbekistan (Table S1). Cotton accessions were sampled from 7362 *G. hirsutum* accessions from different geographical locations preserved at the China National Gene Bank, Cotton Research Institute, Chinese Academy of Agricultural Sciences, Anyang, Henan province. Cluster analysis based on the germplasm database for phenotypic variation, geographic origins, and simple sequence repeat (SSR) data resulted in a base collection of 419 accessions with sufficient genetic diversity for phenotypic traits. The selected accessions represent a core collection of cotton germplasm with ample phenogenetic variation suitable for GWAS.

### 2.2. Optimization of Salt Stress Concentration

A pre-experiment was designed to optimize salt stress concentration; 120 healthy seeds were sterilized to measure relative germination rate (RGR). Six different concentrations of NaCl were set to 0 mM, 100 mM, 150 mM, 200 mM, 250 mM, and 300 mM. For each treatment, seeds were placed/sandwiched in germination boxes (200 × 150 mm diameter) with two moistened germination/filter papers above and below the seeds. Six treatments were applied with two replications of each. 20 mL of distilled water for control and 20 mL of NaCl for treatment were applied. To calculate the relative germination rate (RGR), the number of germinated seeds was recorded after 7 days. Seeds were stated germinated when the size of the radicle was equal or more than half of the seed size. RGR% was calculated as = (number of seeds germinated under stress treatment/number of seeds germinated under control treatment) × 100.

### 2.3. Screening for Salt Tolerance 

A sample of 200 healthy seeds from each genotype was selected and delinted with sulphuric acid followed by surface sterilization with 15% H_2_O_2_ for four hours and rinsed with sterile distilled water for four times to avoid any seed born disease occurrence, subsequently submerged in distilled water for 12 hours. A sample of 120 healthy seeds from each accession was selected and placed in germination boxes (200 × 150 mm diameter), with each containing a double sheet of filter paper soaked in 20 mL (0 mM) distilled water and 20 mL NaCl (150 mM) solution for control and salinity stress, respectively. For the identification of physiological traits, 300 mL volumetric flasks containing 140 g of (sterilized and autoclaved sand) with 25 healthy seeds from each accession were planted and 40 mL of NaCl (150 mM) solution was applied for the salinity stress simulation coupled with controlled replicates as well. A set of 3 independent biological repeats with 3 technical repeats were performed for all 419 accessions. All the seedlings were grown in a phytotron incubating chamber under 14/10 h (light/dark) cycle, 26–28 °C, and 65% relative humidity.

Physiological traits related to salinity tolerance were recorded for assessment, such as fresh weight (FW), seedling length [26], relative water content (RWC), chlorophyll content (ChlC), electric conductivity [27], and malondialdehyde (MDA). After 7 days of seedling growth, salt-tolerant morphophysiological attributes in control and treatment conditions were recorded and their respective ratios were calculated as additional traits. Morphophysiological traits, and their ratio (150_0) as well, for salt tolerance evaluated in this study, both in the control and treatment, included the germination percentage [Ger. % = number of seeds planted/total seeds × 100], relative electric conductivity (REC), MDA—an enol compound that is an imperative marker of oxidative stress tolerance in plants; fresh weight (FW), seedling length/shoot length (SL), relative water content (RWC), and chlorophyll content [25].

### 2.4. Statistical Analysis

Correlation analysis for salinity stress tolerance related traits was performed in R statistical software [28] using the package corrplot [29]. Correlation analysis with the aforementioned package provides a graphical display of the correlation matrix with reordering highly correlated variables in a closed vicinity with an indication of color scale for both positive and negative correlation color patterns. Descriptive statistics were performed with SPSS 22.0 software. 

### 2.5. Factor Analysis

Factor analysis and k-means cluster analysis were performed to evaluate the salt stress tolerance level of 419 accessions, using SPSS software. Kaiser–Meyer–Olkin (KMO) [30] measurements were determined to find the selected variables and suitability of data for factor analysis. A comprehensive evaluation and stratification of salinity stress tolerance were done on the basis of factor scores.

### 2.6. GWAS and SNPs Annotation

Next-generation high throughput Illumina HiSeq platform was used for genome sequencing, which resulted in 6.45 Tb raw sequences with 150 bp read depth. After the sequence quality and filtering process, 6.35 Tb high-quality SNPs were finally used for further analysis. Sequences were aligned and annotated according to the *Gossypium hirsutum* L. genome with Genome Analysis Toolkit (GATK V3.1) and ANNOVAR, respectively, as described by Ma et al. [25]. An association panel of 419 accessions with ∽3.665 million SNPs (MAF ≥ 0.05) was used in GWAS for traits under study. An association study was conducted with efficient mixed-model association eXpedited (EMMAX) software package, and mixed linear model analysis with the following equation was used:

Y = X × *α* + *β* × S + k × *μ* + e

where Y represents phenotype; *α* and *β* are fixed effects representing marker and non-marker effects, respectively; *μ* represents unknown random effects. X, S, and k are the matrices of incidence for *α*, *β,* and *μ*. SNPs were categorized in five regions—exonic, intronic, upstream or downstream, and intergenic—based on their genomic annotation, high-quality, significantly associated SNPs obtained after filtering forged SNPs, and a threshold of −log_10_ (*p*) value ≥ 4. SNPs falling in the coding regions were further classified into synonymous and non-synonymous SNPs, meaning that they either did not cause amino acid change or they caused amino acid change, respectively. 

### 2.7. q-RT PCR of Candidate Genes

To check the compliance of the GWAS results, four salt-tolerant and three salt-sensitive genotypes were chosen for putative gene expression analysis with qRT-PCR. Seedlings were grown in germination boxes (with 3 technical and 3 independent repeats) containing 1 kg of sterilized quartz sand with a 0.3% NaCl content. Data was collected at 7 days after germination (DAG). Root samples were collected at 7 DAG and immediately frozen in liquid nitrogen to stop all biological reactions simultaneously. All samples were subsequently stored at −80 °C for extraction of RNA and synthesis of cDNA. Total cDNA synthesis was accomplished using a PrimeScriptTM RT reagent kit (Perfect Real Time) (TaKaRa, Kyoto, Japan). The qRT-PCR had a final volume of 20 µL, which consisted of 10 µl of SYBR Premix Dimer Eraser (TransTM qRT-PCR Kit, Beijing, China), 2.0 µL of cDNA, 1 µL of primers, and ddH2O. The reactions were amplified at 95 °C for 30 seconds, followed by 40 cycles of 95 °C for 5 seconds, 55 °C for 30 seconds, and 72 °C for 30 seconds. All reactions were executed with three independent biological replications. Gene-specific primers were designed using Oligo7 software.

## 3. Results

### 3.1. Phenotypic Variation in Salt Tolerance Traits

To assess the phenotypic variation seedlings were treated with 150 mM NaCl salt stress and 0 mM as a control. Seven salt tolerance-related traits were measured to explore the level of tolerance: germination rate, relative electrical conductivity, malondialdehyde (MDA) content, fresh weight (FW), shoot length/seedling length (SL), relative water content (RWC), and chlorophyll content. All studied traits showed lower mean values under stress; nevertheless, extreme values of the two traits were high under stress, and five traits had higher extreme values under salt stress conditions than under normal conditions. The coefficient of variation (CV%) of all the traits ranged from 7.18% (RWC_150) to 51.85% (MDA_150) under salt stress conditions (Table 1), while under normal conditions, the CV% ranged from 5.27% (RWC_0) to 36.36% (MDA_0) (Table 2). 

**Figure 1:**
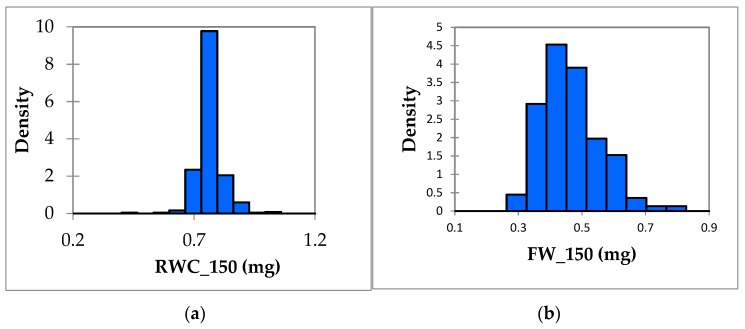
Histogram of relative water content and fresh weight under 150 mM salt stress. (**a**) Normal distribution of RWC_150. (**b**) Normal distribution of FW_150.

When the two results were compared, it can be seen that the higher CV% values under stress conditions, compared to under normal conditions for most of the salt stress-related traits, indicates the presence of wide variation in the natural population comprising 419 accessions under stress conditions. The phenotypes associated with all the traits showed a normal distribution, as shown in (Figure 1), hence, the continuous variation indicates that all the studied traits were quantitative traits, underpinned by multiple genes.

**Figure 2:**
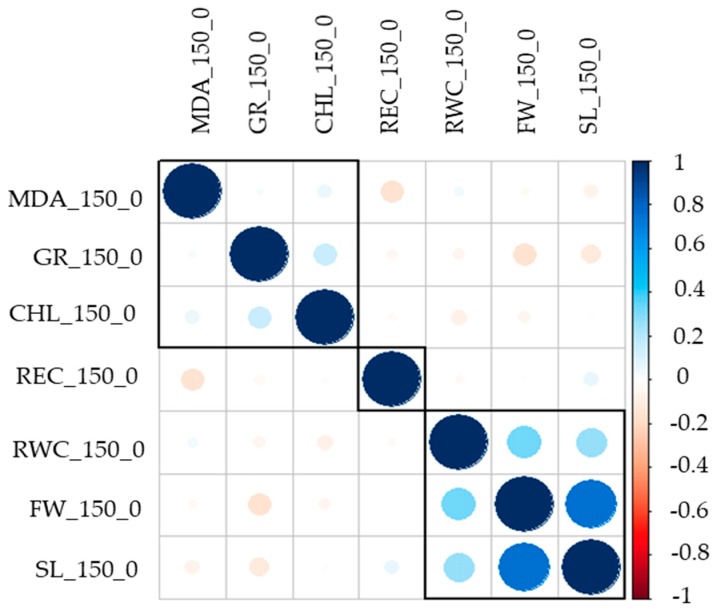
Correllogram shows the correlation among all phenotypic traits color scale shows the strength of the correlation.

The relative values of all the traits were calculated as the ratio of the phenotype under stress and that under normal conditions. The results of the correlational analysis are presented in Figure 2. Figure 2 shows the correlations among the seven traits. It is clearly evident that FW_150_0, SL_150_0, and RWC_150_0 had a highly significant, strong, positive correlation with each other at the 0.0001 level of significance. All the relative values conformed to a Gaussian distribution; Figure 1 shows a Gaussian distribution of RWC_150 and FW_150. The other phenotype distribution is shown in Figure S1. Pearson correlation analysis was used to predict the relationship between the seven salt stress tolerance traits.

### 3.2. Evaluation and Stratification of Salt Tolerance

To assess the salt tolerance level, factor and cluster analyses were performed. The KMO value of 0.656 was higher than 0.5, indicating the suitability of the raw data for factor analysis. A nine-factor solution constituting 63.61% of the cumulative variance was obtained (Table S2). The F factor composite score for salinity stress tolerance stratification of each cotton accession was estimated by six F factors. On the basis of the salt stress tolerance capacity with different F factors, cluster analysis demonstrated that 419 cotton accessions were stratified into four groups. A total of 8, 95, 300 and 16 accessions were stratified into highly tolerant, moderately tolerant, sensitive and highly sensitive to salinity stress, with F factors ranging from 0.89 to 1.27, 0.23 to 0.78, −0.59 to 0.22, and −1.79 to −0.64, respectively (Table S3).

### 3.3. GWAS Analysis of Salt Tolerance-Related Traits

To identify potential candidate genes associated with salt stress tolerance in a natural population of cotton accessions, GWAS was conducted using SNP data and phenotypic data of 7 parameters and their relative values related to salt stress tolerance. Manhattan plots were generated to highlight the significance of SNPs associated with each phenotypic trait. A population tree has already been constructed, and the phylogenetic structure and linkage disequilibrium can be seen from a previous study [25]. In the association analysis of 419 genotypes with low-quality loci (MAF ≥ 0.05; missing rate ≤ 0.2, depth ≥ 3) filtered, the number of spurious SNPs was greatly reduced. Consequently, a final set of 17,264 SNPs having −log_10_ (*p*-value) ≥ 4 was obtained associated with salt stress tolerance phenotypes studied under salinity (150 mM), control (0 mM), and their relative values (150/0). SNPs associated with all parameters and distribution on different genomic regions have been shown in Table 3. 

### 3.4. Identification of Candidate Genes

**Figure 3:**
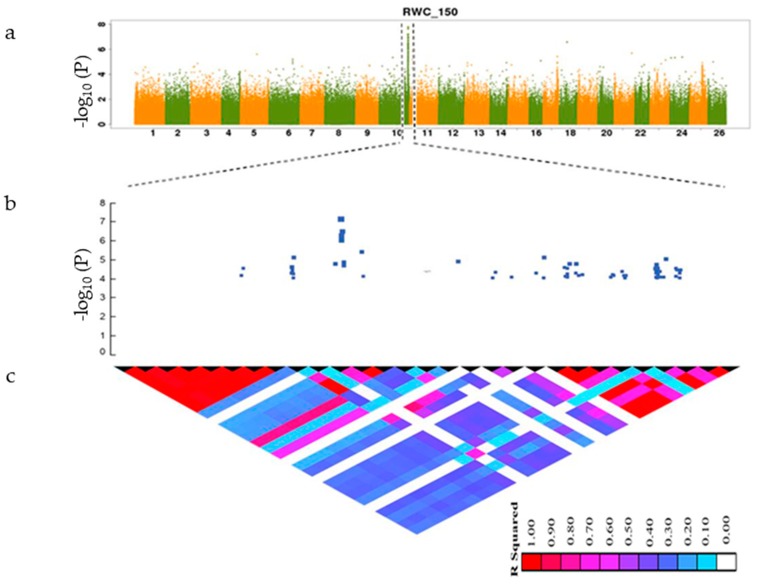
This figure shows the SNPs on all chromosomes and SNPs on chromosome A10 and LD block. (**a**) Manhattan plot of RWC_150. RWC_150 is the value of relative water content under salt stress conditions. X-axis denotes twenty-six chromosomes and y-axis depicts −log_10_ *p*-value; (**b**) local Manhattan plot across the region of chromosome A10 where SNPs were found to be significantly associated with RWC_150; the y-axis is −log_10_ *p*-value; (**c**) linkage disequilibrium block heatmap of RWC_150 triangles shows the r^2^ value of SNPs; the red color indicates 1, the white color indicates 0.

**Figure 4:**
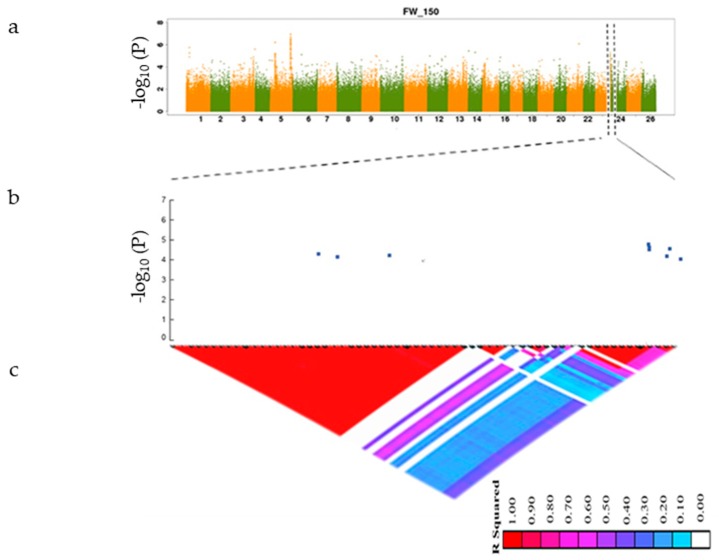
(**a**) Manhattan plot of FW_150. FW_150 is the value of leaf freshwater content under salt stress conditions. The X-axis denotes twenty-six chromosomes and the y-axis depicts −log10 *p*-value; (**b**) local Manhattan plot across the region of chromosome D10 where SNPs were found to be significantly associated with FW_150; the y-axis is −log10 *p*-value; (**c**) linkage disequilibrium block heatmap of FW_150 triangles shows the r^2^ value of SNPs; the red color indicates 1 and the white color indicates 0.

**Figure 5:**
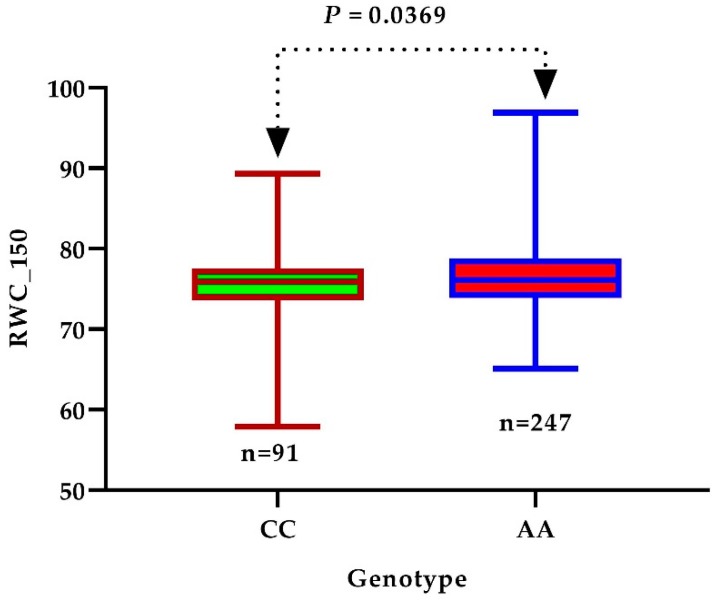
Difference of relative water content, RWC_150, between two haplotypes. In the box plot, the center line shows the median, the box limits are the upper and lower quartiles, and the whisker depicts the range of the data; n shows the number of accessions with the same genotype. Significance of difference (*p*-value) was analyzed with a two-tailed *t* test.

**Figure 6:**
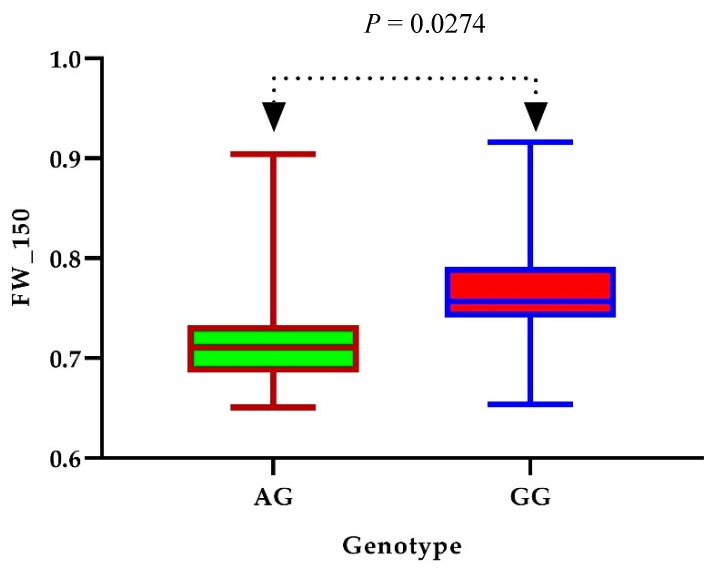
Difference of FW_150 between two haplotypes. In the box plot, the center line shows the median, the box limits are the upper and lower quartiles, and the whisker depicts the range of the data; n shows the number of accessions with the same genotype. Significance of difference (*p*-value) was analyzed with a two-tailed *t* test.

SNPs with −log_10_ (*p*-value) greater than 4 were considered significantly associated with the studied traits. The linkage disequilibrium rate was found to range from 400–500 using the pairwise coefficient of correlation (r^2^) from the maximum value (0.46) to half of the maximum at 742.7 kb for all accessions. The LD decay rate found in our study was higher than that previously reported [31,32,33] (296 kb) but lower than that described by Fang et al. [15] (1000 kb). An LD rate of 26 for cotton was found in a previous study by Ma et al. [25]. The moderate LD decay rate and not highly structured data of the 419 accessions supported the idea that the core collection was appropriate for a GWAS [34]. The annotated genes were searched within the interval associated with the SNP linkage disequilibrium block (LDB) (Figure 3 and Figure 4) and within the physical position of each significant SNP. On the bases of *G.hirsutum* Texas Marker-1 (TM-1) reference genome, we identified a total of 20 putative candidate genes surrounding the peak signals A10_95330133 for RWC_150 on chromosome A10 and D10_61258588 FW_150 on chromosome D10, as shown in Figure 3 and Figure 4. We identified 15 candidate genes surrounding the RWC_150 peak signal, whereas 5 candidate genes were identified for FW_150. Box plots for RWC_150 and FW_150 (Figure 5 and Figure 6) based on haplotypes of two SNPs shows the differences in RWC_150 and FW_150 among the two haplotypes, respectively. The putative candidate genes responsible for the salt stress response were further analyzed and screened using gene expression data of seedlings under 400 mM salt stress for 1, 3, 6, and 12 hours reported by Zhang et al. [35]. The expression profiles of twelve candidate genes were further analyzed with qRT-PCR to check their pattern of expression under salt stress.

### 3.5. Expression Profiles of Presumed Candidate Genes via qRT-PCR Analysis

**Figure 7:**
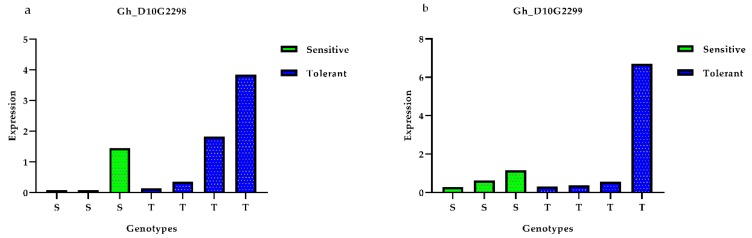

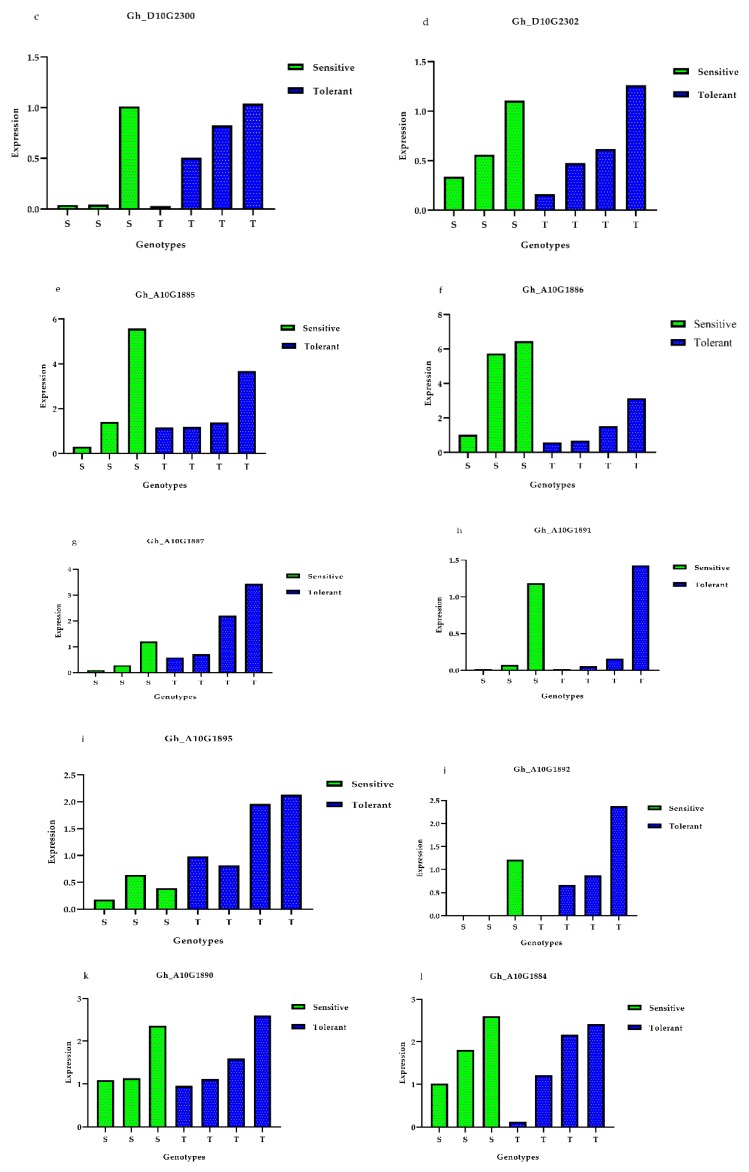
Relative expression of candidate genes by qRT-PCR. These figures (**a**–**l**) shows the relative expression of each selected candidate gene in three salt-sensitive and four salt-tolerant genotypes.

To determine the expression pattern of the twelve genes with gene specific primers (Table S4), four salt-tolerant and three salt-sensitive genotypes were used for qRT-PCR analysis. Compared to those in the salt-sensitive varieties, Gh_D10G2298, Gh_D10G2299, Gh_D10G2302, and Gh_A10G1890 in the salt-tolerant varieties showed a relatively higher expression (Figure 7a,b,d,k), whereas Gh_D10G2300 and Gh_A10G1892 had a very high expression in the salt-tolerant varieties and almost no expression in the salt-sensitive varieties (Figure 7c,j). Gh_A10G1887 had a very high expression in the salt-tolerant varieties compared with the salt-sensitive varieties (Figure 7g); this higher expression further promotes the narrative that these genes were in close association based on salt stress tolerance. However, Gh_A10G1885 and Gh_A10G1886 had a high expression in the salt-sensitive varieties and a low expression in the salt-tolerant varieties (Figure 7e,f).

## 4. Discussion

In the current study, we performed a GWAS of salinity stress tolerance traits at the seedling stage with a core collection of 419 cotton accessions selected from genetically diverse backgrounds and SNPs from the high-throughput Illumina sequencing platform. The findings of this study complement the understanding of the complex nature of salt stress tolerance mechanisms and the scouring of novel alleles and candidate genes. One of the implications of the present study is the possibility of accelerating the progress of cotton stress tolerance breeding.

Salt stress tolerance is a complex trait regulated by polygenes [36]. GWASs provide an opportunity to explore genes responsible for quantitative trait variation in plants and animals [37]. Relative to forward genetic approaches, GWASs have the potential to identify genes with smaller phenotypic effects [38]. GWASs have become an obvious general methodology for studying the effects of natural variations and traits of agricultural and economic importance [39]. A handful of research papers are available on association studies, particularly on fiber, yield, disease and their respective component traits, in cotton and several other crop species, but little work has been done on association analyses for stress tolerance in general in other crop species, particularly cotton [15,16,17,40]. To date, few studies have investigated salt stress tolerance with natural variation and genome-wide markers by means of GWAS approaches. Jia et al. [41] identified three SSR markers associated with salt tolerance by employing a mixed linear model and a panel of 323 cotton accessions and using 106 SSR markers. Using 179 polymorphic SSR markers in 503 upland cotton accessions, the researchers identified 15 SSR and 3119 SNP markers associated with relative germination rate under salt stress and ultimately found four differentially expressed candidate genes in tolerant and sensitive accessions under salt stress. Sun et al. [21], screened 713 accessions and identified 23 SNPs representing seven genomic regions that were significantly associated with salt tolerance level (STL) and relative survival rate (RSR). Furthermore, 280 putative genes showing different expression levels were screened, and six apparent putative genes were validated with qRT-PCR in salt-tolerant and sensitive varieties.

Lectin receptor-like kinases (LecRKS) play an important role in plant innate defense mechanisms. L-type LecRKs, one of the three types of LecRKs, are considered to play an important role in abiotic stress signaling in *Arabidopsis* [42]. In our study of fresh weight under salt stress, we found that the expression of the RNA of Gh_D10G2298, which encodes an LeCRK, under salt stress was high in salt-tolerant genotypes compared to salt-sensitive genotypes, as shown in Figure 7. Rubisco activase (RCA) is an important enzyme involved in the carboxylation and oxygenation of ribulose-1,5-bisphosphate carboxylase/oxygenase (Rubisco) and participates in the photosynthetic carbon reduction cycle. In a study conducted by Chen et al. [43], rubisco activase responded to abiotic stress in multiple ways. An investigation was carried out with respect to the RCA gene’s 2.0 kb 5′-upstream promoter region, some cis-elements related to certain stress-related components were identified in the RCA promoter. Multiple species comparisons with respect to the RCA protein revealed conserved regions among different species; their extent and nature varied. This finding might reveal the various transcription and translation splicing stages of the two RCA isoforms during adaptation to various abiotic stresses. These findings suggest that RCA, particularly RCAL, is a multiple responder to abiotic stresses.

In our study of leaf fresh weight under salinity stress, the gene Gh_D10G2299 on chromosome D10 was found to encode (RCA2) protein. This protein has gained much attention as a regulator of a number of biotic and abiotic stress tolerances. Bi et al. [44] studied the overexpression of rubisco activase in cucumber (*Cucumis sativus* L.); it was found that CsRCA overexpression resulted in increased leaf area, plant height, and dry matter content, with a reduced root/shoot ratio in transgenic cucumber plants, compared to wild-type plants. 

Salinity stress causes all types of root damage in all crops. A study conducted by Robin et al. [45] showed that adventitious root length and density of wheat crops decreased by 25% and 40%, respectively, under salt stress. Lateral organ boundary (LOB) proteins are expressed in lateral and adventitious roots in plants [45]. Interestingly, we found the lateral organ boundary protein-coding gene, Gh_D10G2300, on chromosome D10 under salt stress. The relative expression of Gh_D10G2300 under salt stress, as shown in Figure 7, is higher in salt-tolerant genotypes than in salt-sensitive genotypes. Chlorophyll contents are reduced under salt stress, as shown in our study, so if chloroplast performance is improved, plant performance could increase under salt stress. In our study, we found that the Gh_D10G2302 gene, which encodes a 15 kDa thylakoid lumen protein, can enhance stress tolerance in crop plants. Chaperones and chaperonins play an important role in nascent protein folding, stabilization, and assistance to obtain a particular function [46]. In a study conducted by Rodríguez et al. [47], PFD5, a chaperone protein, was found to play an important role in *Arabidopsis thaliana* L. salt stress tolerance. We found a gene for RWC_150 on chromosome A10, Gh_A10G1885, coding for a probable prefoldin subunit 2 chaperone. Differential gene expression was detected between the salt-tolerant genotypes and salt-sensitive genotypes under salt stress. Splicing factor 3B subunit 3 belongs to the ion channel family and participates in RNA modulation in plants, which involves an inverse resistance response in plants [48]. In our study, the gene Gh_A10G1886, which encodes the splicing factor 3b subunit3 SF3B3, was found on chromosome A10 in RWC_150. Its relative expression was high in salt-sensitive genotypes and vice versa. Dirigent and dirigent-like family (DIR) proteins are a group of proteins responsible for lignification, pathogen infection responses, and abiotic stress tolerance in plants. DIR genes play a vital role in augmenting stress tolerance in different crop species. Yang et al. [49], studied a dirigent-like gene in sugarcane designated ScDir with a full-length cDNA sequence. The expression of ScDir in an *E. coli* system indicated that ScDir protein improved the host cell’s tolerance to polyethylene glycol (PEG) and NaCl. The ScDir expression level increased in sugarcane seedlings under H_2_O_2_, PEG, and NaCl stress. ScDir expression was significantly upregulated under PEG stress, and the highest level of expression was observed at 12 hours post-stress application. Thus, both the ScDir-hosted cell performance and the enhanced expression in sugarcane suggest that the ScDir gene provides responses to abiotic stresses, such as drought, salt, and oxidation.

Salinity stress induces osmolyte wavering in plant cells, consequently causing relative water imbalance. Relative water content (RWC) is considered the most suitable sign of plant water status in terms of the physiological concern of cellular water scarcity under water deficit and salt stress [50].

With respect to relative water content under salt stress (RWC_150), a group of three dirigent genes, Gh_A10G1887, Gh_A10G1888, and Gh_A10G1889, was found: two genes (Gh_A10G1887 and Gh_A10G1889) coding for dirigent protein 25 and one gene (Gh_A10G1888) coding for dirigent protein 9 on chromosome A10. These speculative genes were homologous to *Arabidopsis* At1g07730.2 and At2g39430.1, which encode members of the disease resistance protein family.

A study conducted by Xu et al. [51] confirmed the role of the glutathione S-transferase gene (GST) in genetically modified tobacco under drought and salt stress. Genetic transformation of the glutathione S-transferase gene GsGST from wild soybean (*Glycine soja* L.) enhanced drought and salt tolerance in transgenic tobacco. Tobacco plants overexpressing the GsGST gene showed a six-fold increase of GST expression compared with that of wild-type (WT) plants, further revealing improved desiccation resistance and higher tolerance to salt and mannitol at the seedling stage than WT plants, as corroborated by longer root length and less growth obstruction in the former. Kumar et al. [52] studied the role of a member of the lambda class of proteins, OsGSTL2, by checking the expression in a heterologous system—*Arabidopsis*. Transgenic lines were analyzed to check their response to a number of abiotic stresses, such as heavy metal, cold, osmotic and salt stresses. Differential expression of OsGSTL genes was observed in arsenate-sensitive and arsenate-tolerant rice accessions. Heterologous expression of glutathione S-transferase gene 2 in *Arabidopsis* provided tolerance to different heavy metal, salt, drought and other abiotic stresses during early germination stages.

On chromosome A10, we found Gh_A10G1891, a DHAR2 gene that is homologous to the *Arabidopsis* gene AT1G75270.1 and shares 76% identity with the encoded glutathione s-transferase DHAR2 protein. Glutathione s-transferases (GSTs) are thought to play major roles in oxidative stress metabolism. A number of studies have confirmed their role in stress tolerance.

Plant scientists consider sulfur an important constituent in plants to withstand abiotic stress [53]. The level of sulfate in the xylem acts as a signal for abscisic acid-dependent leaf stomatal closure during the early onset stage of drought when ABA synthesis is limited to the leaves [54]. Sulfur metabolism and ABA biosynthesis together ensure sufficient cysteine for ABA production under abiotic stress. Sulfate acts as a precursor of cysteine, which plays a crucial role in ABA synthesis. Gallardo et al. [55], conducted a comparative study of the SULTR gene family under drought and salinity stress in *Arabidopsis* and *Medicago truncatula*. The SULTR genes in *M. truncatula* were found to be similarly regulated, as in *Arabidopsis*, they likely encode factors for improving sulfate transport dimensions. Group 3 SULTR genes were found to be abiotic stress-responsive genes common between *Arabidopsis* and *M. truncatula* [56].

Metal toxicity produces reactive oxygen species (ROS) in plants, leading to an imbalance in cell homeostasis, breakage of the DNA, protein denaturation, and damage to the cell membrane and photosynthetic machinery, leading to cell death [57,58]. Plant metal tolerance proteins (MTPs) are divalent-cation/H+ antiporters and generally act to efflux metals from the cytoplasm [59]. We found a probable role of the metal tolerance protein-coding gene Gh_A10G1895 in RWC_150; its expression was high in the salt-tolerant genotypes compared to the salt stress-sensitive genotypes. Therefore, this gene may play a vital role in water homeostasis. We also found two genes Gh_A10G1884 and Gh_A10G1890 that have no previously studied role for salt stress tolerance in any crop. Therefore, functional studies of these two genes may provide useful insights into their role in salinity stress tolerance in cotton. 

## 5. Conclusions

A core collection of 419 accessions with diverse genetic backgrounds had large phenotypic and genotypic variations for almost all studied traits associated with salt tolerance. A total of 23 SNPs showing significant association with the different traits were identified. We further selected two SNPs associated with FW_150 and RWC_150 as the most suitable SNPs, as they have high p and r^2^ values. Genes detected within the LD block of the candidate SNPs were considered candidate genes. We further validated 12 putative genes using four salt-tolerant and three salt-sensitive varieties under salt stress by qRT-PCR. Our study has provided useful reference information about candidate loci and genes that could be useful for future cotton salt tolerance breeding programs.

## Figures and Tables

**Table 1:** Descriptive statistics of phenotypes under normal conditions.

Variable	Minimum	Maximum	Mean	Std. Deviation	CV%	Skewness (Pearson)	Kurtosis (Pearson)
GR_0	22	100	80.676	15.48	19.187	–1.746	4.404
REC_0	0.002	0.87	0.476	0.102	21.428	–0.509	4.814
MDA_0	0.001	0.093	0.033	0.012	36.363	0.913	3.028
FW_0	0.369	1.07	0.577	0.087	15.077	0.765	2.245
SL_0	4.84	12.667	7.559	1.068	14.128	0.417	1.018
RWC_0	0.561	1.06	0.91	0.048	5.2747	–1.913	13.762
CHL_0	26.492	87.053	50.665	4.851	9.5746	0.535	9.043

Table 1 phenotypic traits abbreviated as GR, germination; REC, relative electric conductivity; MDA, melondialdehyde; FW, fresh weight; SL, seedling length; RWC, relative water content; CHL, chlorophyll content; 0, (0 mM).

**Table 2:** Descriptive statistics of phenotypes under salt stress.

Variable	Minimum	Maximum	Mean	Std. Deviation	CV%	Skewness (Pearson)	Kurtosis (Pearson)
GR_150	13.33	98	74.209	16.152	21.765	−1.262	1.083
REC_150	0.426	3.057	1.083	0.319	29.455	2.291	9.09
MDA_150	0.001	0.111	0.027	0.014	51.851	1.258	3.633
FW_150	0.28	0.818	0.464	0.092	19.827	0.773	0.567
SL_150	2.487	8.91	5.801	1.134	19.548	0.211	−0.43
RWC_150	0.434	1.054	0.766	0.055	7.180	0.51	7.185
CHL_150	25.74	60.933	48.348	5.571	11.522	−0.572	0.724

Table 2. phenotypic traits abbreviated as GR, germination; REC, relative electric conductivity; MDA, melondialdehyde; FW, fresh weight; SL, seedling length; RWC, relative water content; CHL, chlorophyll content; 150, (150mM).

**Table 3:** Distribution of single-nucleotide polymorphisms (SNPs) on the gene regions.

Trait	Downstream	Exonic	Intergenic	Intronic	Splicing	Upstream	Upstream; Downstream	Total
CHL_0	18	34	115	39		37	9	252
CHL_150	13	11	228	9		21	4	286
CHL_150_0	8	4	124	6		2	3	147
FW_0	534	221	5179	1607	1	507	117	8166
FW_150	15	17	140	28		53	14	267
FW_150_0	8	5	184	13		11	4	225
GR_0	10	18	253	13		9	7	310
GR_150	7		125	1		3	1	137
GR_150_0	2	1	74	6		8	1	92
MDA_0	23	4	199	17		16	8	267
MDA_150	14	11	210	12		31	5	283
MDA_150_0	11	5	122	6		12	4	160
REC_0	31	23	332	41		30	17	474
REC_150	50	41	259	32		45	18	445
REC_150_0	268	140	2294	319	1	343	78	3443
RWC_0	33	9	568	33		39	8	690
RWC_150	30	8	217	30		24	12	321
RWC_150_0	17	6	368	11		15	10	427
SL_0	10	4	125	13	1	8	1	162
SL_150	11	10	103	15		21	14	174
SL_150_0	28	16	412	20		48	12	536
Grand Total	1141	588	11,631	2271	3	1283	347	17,264

REC_150_0 had the highest number (3443) of SNPs and RGR_150_0 had the lowest number (92) of SNPs. Widespread association and distribution of SNPs with different traits and genomic regions depict an intricate genetic basis of salt stress tolerance. Out of 23 highly associated SNPs, we finally selected 2 candidate SNPs in compliance with high −log_10_ (*p*-value) and r^2^ value, A10_95330133 associated with RWC_150 on chromosome A10 and D10_61258588 associated with FW_150 on chromosome D10. The quantile–quantile (QQ) plot of expected and observed *p*-values was outlined and SNPs that had deviated p-values from the linear showed reasonable positives (Figure S2) and indicated that the EMMAX model was optimal and could be used to scour association signals.

## Supplementary Materials

The following are available online at https://www.mdpi.com/2073-4425/10/10/829/s1, Figure S1: Gaussian distribution, Figure S2: Quantile Quantile plots, Table S1: list of cotton accessions used in this study, Table S2: cumulative variance, Table S3: factor scores, Table S4: List of primer sequences.

## References

[1] Zhang H., Han B., Wang T., Chen S., Li H., Zhang Y., Dai S. (2011). Mechanisms of plant salt response: Insights from proteomics. J. Proteome Res..

[2] Stewart J.M., Oosterhuis D., Heitholt J.J., Mauney J.R. (1986). Cotton Physiology.

[3] Wang J., Huang X., Zhong T., Chen Z. (2011). Review on Sustainable Utilization of Salt-affected Land. Acta Geogr. Sin..

[4] Zhu J.-K. (2002). Salt and drought stress signal transduction in plants. Annu. Rev. Plant Biol..

[5] Munns R., Tester M. (2008). Mechanisms of salinity tolerance. Annu. Rev. Plant Biol..

[6] Goossens R., Van Ranst E. (1998). The use of remote sensing to map gypsiferous soils in the Ismailia Province (Egypt). Geoderma.

[7] Gilland B.J. (2002). World population and food supply: Can food production keep pace with population growth in the next half-century?. Food Policy.

[8] Brachi B., Morris G.P., Borevitz J.O. (2011). Genome-wide association studies in plants: The missing heritability is in the field. Genome Biol..

[9] Korte A., Farlow A. (2013). The advantages and limitations of trait analysis with GWAS: A review. Plant Methods.

[10] Borevitz J.O., Nordborg M. (2003). The impact of genomics on the study of natural variation in Arabidopsis. Plant Physiol..

[11] Ogura T., Busch W. (2015). From phenotypes to causal sequences: Using genome wide association studies to dissect the sequence basis for variation of plant development. Curr. Opin. Plant Biol..

[12] Pantaliao G.F., Narciso M., Guimares C., Castro A., Colombari J.M., Breseghello F., Rodrigues L., Vianello R.P., Borba T.O., Brondani C. (2016). Genome wide association study (GWAS) for grain yield in rice cultivated under water deficit. Genetica.

[13] Li D., Dossa K., Zhang Y., Wei X., Wang L., Zhang Y., Liu A., Zhou R., Zhang X. (2018). GWAS Uncovers Differential Genetic Bases for Drought and Salt Tolerances in Sesame at the Germination Stage. Genes.

[14] Chao D.-Y., Silva A., Baxter I., Huang Y.S., Nordborg M., Danku J., Lahner B., Yakubova E., Salt D.E. (2012). Genome-wide association studies identify heavy metal ATPase3 as the primary determinant of natural variation in leaf cadmium in Arabidopsis thaliana. PLoS Genet..

[15] Fang L., Wang Q., Hu Y., Jia Y., Chen J., Liu B., Zhang Z., Guan X., Chen S., Zhou B. (2017). Genomic analyses in cotton identify signatures of selection and loci associated with fiber quality and yield traits. Nat. Genet..

[16] Li T., Ma X., Li N., Zhou L., Liu Z., Han H., Gui Y., Bao Y., Chen J., Dai X. (2017). Genome-wide association study discovered candidate genes of Verticillium wilt resistance in upland cotton (*Gossypium hirsutum* L.). Plant Biotechnol. J..

[17] Sun Z., Wang X., Liu Z., Gu Q., Zhang Y., Li Z., Ke H., Yang J., Wu J., Wu L. (2017). Genome-wide association study discovered genetic variation and candidate genes of fibre quality traits in *Gossypium hirsutum* L.. Plant Biotechnol. J..

[18] Sun H., Meng M., Yan Z., Lin Z., Nie X., Yang X. (2019). Genome-wide association mapping of stress-tolerance traits in cotton. Crop J..

[19] Cai C., Zhu G., Zhang T., Guo W. (2017). High-density 80 K SNP array is a powerful tool for genotyping G. hirsutum accessions and genome analysis. BMC Genom..

[20] Diouf L., Pan Z., He S.-P., Gong W.-F., Jia Y.H., Magwanga R.O., Romy K.R.E., Rashid H.O., Kirungu J.N., Du X. (2017). High-Density Linkage Map Construction and Mapping of Salt-Tolerant QTLs at Seedling Stage in Upland Cotton Using Genotyping by Sequencing (GBS). Int. J. Mol. Sci..

[21] Sun Z., Li H., Zhang Y., Li Z., Ke H., Wu L., Zhang G., Wang X., Ma Z. (2018). Identification of SNPs and Candidate Genes Associated With Salt Tolerance at the Seedling Stage in Cotton (*Gossypium hirsutum* L.). Front. Plant Sci..

[22] Lacape J.-M., Nguyen T.-B., Courtois B., Belot J.-L., Giband M., Gourlot J.-P., Gawryziak G., Roques S., Hau B. (2005). QTL analysis of cotton fiber quality using multiple *Gossypium hirsutum* × *Gossypium barbadense* backcross generations. Crop Sci..

[23] Chartzoulakis K., Klapaki G. (2000). Response of two greenhouse pepper hybrids to NaCl salinity during different growth stages. Sci. Hortic..

[24] Sexton P., Gerard C. (1982). Emergence Force of Cotton Seedlings as Influenced by Salinity 1. Agron. J..

[25] Ma Z., He S., Wang X., Sun J., Zhang Y., Zhang G., Wu L., Li Z., Liu Z., Sun G. (2018). Resequencing a core collection of upland cotton identifies genomic variation and loci influencing fiber quality and yield. Nat. Genet..

[26] Maqbool M.A., Aslam M., Ali H. (2017). Breeding for improved drought tolerance in Chickpea (*Cicer arietinum* L.). Plant Breed..

[27] Kaashyap M., Ford R., Kudapa H., Jain M., Edwards D., Varshney R., Mantri N. (2018). Differential regulation of genes involved in root morphogenesis and cell wall modification is associated with salinity tolerance in chickpea. Sci. Rep..

[28] R Core Team (2017). R: A Language and Environment for Statistical Com—Putting.

[29] Wei T., Simko V. (2017). R Package “Corrplot”: Visualization of a Correlation Matrix.

[30] Cerny B.A., Kaiser H.F. (1977). A Study Of A Measure Of Sampling Adequacy for Factor-Analytic Correlation. Multivar. Behav. Res..

[31] Wang K., Wang Z., Li F., Ye W., Wang J., Song G., Yue Z., Cong L., Shang H., Zhu S. (2012). The draft genome of a diploid cotton *Gossypium raimondii*. Nat. Genet..

[32] Wang M., Tu L., Lin M., Lin Z., Wang P., Yang Q., Ye Z., Shen C., Li J., Zhang L. (2017). Asymmetric subgenome selection and cis-regulatory divergence during cotton domestication. Nat. Genet..

[33] Said J.I., Lin Z., Zhang X., Song M., Zhang J. (2013). A comprehensive meta QTL analysis for fiber quality, yield, yield related and morphological traits, drought tolerance, and disease resistance in tetraploid cotton. BMC Genom..

[34] Yano K., Yamamoto E., Aya K., Takeuchi H., Lo P.C., Hu L., Yamasaki M., Yoshida S., Kitano H., Hirano K. (2016). Genome-wide association study using whole-genome sequencing rapidly identifies new genes influencing agronomic traits in rice. Nat Genet.

[35] Zhang T., Hu Y., Jiang W., Fang L., Guan X., Chen J., Zhang J., Saski C.A., Scheffler B.E., Stelly D.M. (2015). Sequencing of allotetraploid cotton (*Gossypium hirsutum* L. acc. TM-1) provides a resource for fiber improvement. Nat. Biotechnol..

[36] Flowers T.J. (2004). Improving crop salt tolerance. J. Exp. Bot..

[37] Mackay T.F., Stone E.A., Ayroles J.F. (2009). The genetics of quantitative traits: Challenges and prospects. Nat. Rev. Genet..

[38] Gao L., Turner M.K., Chao S., Kolmer J., Anderson J.A. (2016). Genome Wide Association Study of Seedling and Adult Plant Leaf Rust Resistance in Elite Spring Wheat Breeding Lines. PLoS ONE.

[39] Atwell S., Huang Y.S., Vilhjalmsson B.J., Willems G., Horton M., Li Y., Meng D., Platt A., Tarone A.M., Hu T.T. (2010). Genome-wide association study of 107 phenotypes in *Arabidopsis thaliana* inbred lines. Nature.

[40] Su J., Fan S., Li L., Wei H., Wang C., Wang H., Song M., Zhang C., Gu L., Zhao S. (2016). Detection of Favorable QTL Alleles and Candidate Genes for Lint Percentage by GWAS in Chinese Upland Cotton. Front. Plant Sci..

[41] Jia Y.-H., Sun J.-L., Wang X.-W., Zhou Z.-L., Pan Z.-E., He S.-P., Pang B.-Y., Wang L.-R., Du X.-M. (2014). Molecular Diversity and Association Analysis of Drought and Salt Tolerance in *Gossypium hirsutum* L. Germplasm. J. Integr. Agric..

[42] Garcia-Hernandez M., Berardini T., Chen G., Crist D., Doyle A., Huala E., Knee E., Lambrecht M., Miller N., Mueller L.A. (2002). TAIR: A resource for integrated Arabidopsis data. Funct. Integr. Genom..

[43] Chen Y., Wang X.-M., Zhou L., He Y., Wang D., Qi Y.-H., Jiang D.-A. (2015). Rubisco Activase Is Also a Multiple Responder to Abiotic Stresses in Rice. PLoS ONE.

[44] Bi H., Liu P., Jiang Z., Ai X. (2017). Overexpression of the rubisco activase gene improves growth and low temperature and weak light tolerance in *Cucumis sativus* L.. Physiol. Plant..

[45] Robin A.H.K., Matthew C., Uddin M.J., Bayazid K.N. (2016). Salinity-induced reduction in root surface area and changes in major root and shoot traits at the phytomer level in wheat. J. Exp. Bot..

[46] Cao J. (2016). Analysis of the Prefoldin Gene Family in 14 Plant Species. Front. Plant Sci..

[47] Rodríguez-Milla M.A., Salinas J. (2009). Prefoldins 3 and 5 Play an Essential Role in Arabidopsis Tolerance to Salt Stress. Mol. Plant.

[48] Chen X., Hao L., Pan J., Zheng X., Jiang G., Jin Y., Gu Z., Qian Q., Zhai W., Ma B. (2012). SPL5, a cell death and defense-related gene, encodes a putative splicing factor 3b subunit 3 (SF3b3) in rice. Mol. Breed..

[49] Yang X., Liang Z., Wen X., Lu C. (2008). Genetic engineering of the biosynthesis of glycinebetaine leads to increased tolerance of photosynthesis to salt stress in transgenic tobacco plants. Plant Mol. Biol..

[50] Tahara M., Carver B.F., Johnson R.C., Smith E.L. (1990). Relationship between relative water content during reproductive development and winter wheat grain yield. Euphytica.

[51] Xu J., Xing X.-J., Tian Y.-S., Peng R.-H., Xue Y., Zhao W., Yao Q.-H. (2015). Transgenic Arabidopsis Plants Expressing Tomato Glutathione S-Transferase Showed Enhanced Resistance to Salt and Drought Stress. PLoS ONE.

[52] Kumar S., Asif M.H., Chakrabarty D., Tripathi R.D., Dubey R.S., Trivedi P.K. (2013). Expression of a rice Lambda class of glutathione S-transferase, OsGSTL2, in Arabidopsis provides tolerance to heavy metal and other abiotic stresses. J. Hazard. Mater..

[53] Chan K.X., Wirtz M., Phua S.Y., Estavillo G.M., Pogson B.J. (2013). Balancing metabolites in drought: The sulfur assimilation conundrum. Trends Plant Sci..

[54] Ernst L., Goodger J.Q., Alvarez S., Marsh E.L., Berla B., Lockhart E., Jung J., Li P., Bohnert H.J., Schachtman D.P. (2010). Sulphate as a xylem-borne chemical signal precedes the expression of ABA biosynthetic genes in maize roots. J. Exp. Bot..

[55] Gallardo K., Courty P.-E., Le Signor C., Wipf D., Vernoud V. (2014). Sulfate transporters in the plant’s response to drought and salinity: Regulation and possible functions. Front. Plant Sci..

[56] Hyung D., Lee C., Kim J.-H., Yoo D., Seo Y.-S., Jeong S.-C., Lee J.-H., Chung Y., Jung K.-H., Cook D.R. (2014). Cross-family translational genomics of abiotic stress-responsive genes between Arabidopsis and Medicago truncatula. PLoS ONE.

[57] Schutzendubel A., Polle A. (2002). Plant responses to abiotic stresses: Heavy metal-induced oxidative stress and protection by mycorrhization. J. Exp. Bot..

[58] Flora S.J. (2009). Structural, chemical and biological aspects of antioxidants for strategies against metal and metalloid exposure. Oxidative Med. Cell. Longev..

[59] Gustin J.L., Zanis M.J., Salt D.E. (2011). Structure and evolution of the plant cation diffusion facilitator family of ion transporters. BMC Evol. Biol..

